# Flexible and Wavelength-Selective MoS_2_ Phototransistors with Monolithically Integrated Transmission Color Filters

**DOI:** 10.1038/srep40945

**Published:** 2017-01-18

**Authors:** Geonwook Yoo, Sol Lea Choi, Sang Jin Park, Kyu-Tae Lee, Sanghyun Lee, Min Suk Oh, Junseok Heo, Hui Joon Park

**Affiliations:** 1 School of Electronic Engineering, Soongsil University, Seoul, 06938, South Korea; 2 Display Materials & Components Research Center, Korea Electronics Technology Institute, Gyeonggi 13509, South Korea; 3 Department of Energy Systems Research, Ajou University, Suwon 16499, South Korea; 4 Department of Materials Science and Engineering, University of Illinois, Urbana-Champaign, Illinois 61801, USA; 5 Department of Electrical and Computer Engineering, Ajou University, Suwon 16499, South Korea

## Abstract

Color-selective or wavelength-tunable capability is a crucial feature for two-dimensional (2-D) semiconducting material-based image sensor applications. Here, we report on flexible and wavelength-selective molybdenum disulfide (MoS_2_) phototransistors using monolithically integrated transmission Fabry-Perot (F-P) cavity filters. The fabricated multilayer MoS_2_ phototransistors on a polyarylate substrate exhibit decent electrical characteristics (*μ*_*FE*_ > 64.4 cm^2^/Vs, on/off ratio > 10^6^), and the integrated F-P filters, being able to cover whole visible spectrum, successfully modulate the spectral response characteristics of MoS_2_ phototransistors from ~495 nm (blue) to ~590 nm (amber). Furthermore, power dependence of both responsivity and specific detectivity shows similar trend with other reports, dominated by the photogating effect. When combined with large-area monolayer MoS_2_ for optical property enhancement and array processing, our results can be further developed into ultra-thin flexible photodetectors for wearables, conformable image sensor, and other optoelectronic applications.

The recent advent of two-dimensional (2-D) semiconducting materials, such as transition metal dichalcogenides (TMDCs), has gained great interest due to their intriguing electrical and optical properties^1^,^2^,^3^,^4^,^5^,^6^, and thus they have been considered as a promising candidate for emerging electronic and optoelectronic devices^3^,^7^,^8^,^9^,^10^,^11^,^12^. As for optoelectronics, in particular, they attain great potential due to their controllable bandgap energy depending on layer thickness and its high absorptivity^2^,^13^. The existing bandgap allows low-dark current by completely turning off the channel, which is favorable for highly sensitive photoresponse. Furthermore, curved, flexible and even conformable photodetectors for wearable and light-weight device applications can be envisioned by considering outstanding mechanical properties of the 2-D layered materials^4^,^12^,^14^,^15^,^16^. The recent advances in scalable and large-area process of TMDCs (e.g. MoS_2_, MoSe_2_) make these merits more compelling and even demand implementation and integration toward image sensor^17^,^18^,^19^,^20^,^21^,^22^. So far, many studies were mainly devoted to enhance figure-of-merits (e.g. responsivity and specific detectivity) of TMDCs-based photodetectors by adopting a surface plasmonic nanostructure^23^, a unique device structure^24^, and various organic and inorganic over-layers^25^,^26^,^27^,^28^, However, color-selectivity or wavelength tunable capability in visible range is another essential feature required for image sensor applications^29^,^30^. Yet, for our best knowledge, none of results has been reported on color-selectivity of TMDCs-based photodetector by integrating color-filters.

Here, we present flexible and wavelength-selective MoS_2_ phototransistors using monolithically integrated transmission Fabry-Perot (F-P) cavity filters, which can modulate its spectral response from blue (~495 nm) to amber (~590 nm) color. We designed and integrated the F-P cavity color-filters based on a metal-insulator-metal (MIM) structure^31^,^32^,^33^,^34^,^35^, instead of traditional organic dye-based filters that are susceptible to environment and difficult for making ultra-thin structure^36^,^37^. Electrical characteristics before and after the F-P cavity integration and spectral response characteristics as well as other figure-of-merits (e.g. responsivity, specific detectivity) of the integrated MoS_2_ phototransistors are investigated and discussed. The results unveil the potential of MoS_2_ phototransistors integrated with thin F-P cavity color filters for applications in flexible and non-planar image sensors.

## Results and Discussion

**Figure 1:**
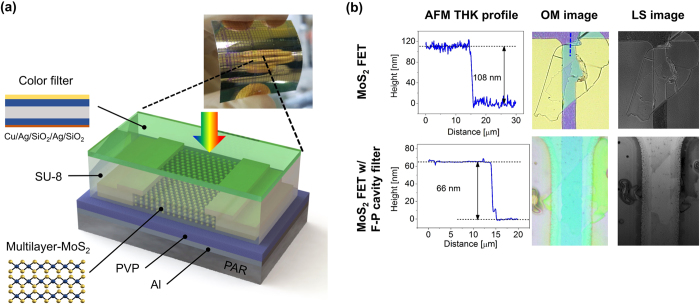
(**a**) Schematic illustration of the fabricated MoS_2_ phototransistor integrated with Fabry-Perot cavity filter and photograph image of the bent device on a PAR substrate. (**b**) The measured thickness of MoS_2_ flakes by atomic force microscope (AFM), optical microscope (OM), and laser scanning (LS) images of a bare MoS_2_ phototransistor (bare-MoS_2_) and MoS_2_ phototransistor with F-P cavity filter for amber color are shown on the top-row and on the bottom-row, respectively. The atomic layer numbers corresponding to the MoS_2_ thickness were about 166 and 102 layers, respectively, assuming monolayer thickness of 6.5 Å.

A 3-dimensional illustration of the multilayer MoS_2_ phototransistor integrated with Fabry–Perot (F-P) cavity color filter on a flexible plastic substrate is depicted in Fig. 1a. First, back-gated MoS_2_ FETs using Poly(4-vinylphenol) PVP gate dielectric were fabricated on a polyarylate (PAR) film using a conventional mechanical exfoliation method. Process temperature was controlled below 150 °C to avoid thermal stress onto PAR film and adhesive layer. After that, SU-8 was spin-coated with a target thickness of about 2.5 um for optical separation of MoS_2_ phototransistor from the F-P cavity, followed by S/D contact opening using exposure and develop process. F-P cavity structures were fabricated by sequentially depositing Ag, SiO_2_ and Ag on SU-8 layer using e-beam evaporator, and S/D contacts were opened again using conventional lithography and wet etching process. Before Ag layer deposition, ultrathin Cu seed layer (1 nm) was deposited using E-beam evaporator to improve wettability of the thin Ag film on the SU-8 layer minimizing the light scattering loss induced from a rough surface^38^. In addition, SiO_2_ capping layer (10 nm) was deposited on Ag/SiO_2_/Ag F-P cavity to protect the top Ag layer from oxidation during S/D contact opening process. A bending image of the flexible MoS_2_ phototransistors with F-P cavity is also shown in Fig. 1a. Figure 1b shows atomic force microscope (AFM) thickness profiles, optical microscopy (OM), and laser scanning (LS) images of the integrated devices; a bare MoS_2_ phototransistor (bare-MoS_2_) is on the top-row, and MoS_2_ phototransistor with amber color filter (MoS_2_-amber) is on the bottom-row. Although bare-MoS_2_ could be identified using both OM and LS methods, MoS_2_ channel area of the MoS_2_-amber was hardly recognized due to visible light filtering and thus LS was used to locate the devices with the F-P filter.

**Figure 2:**
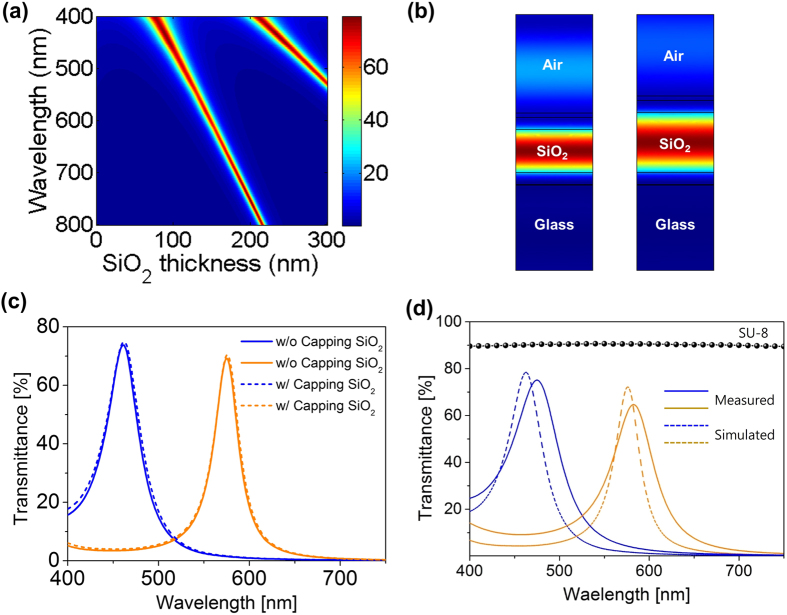
(**a**) Calculated transmission of the Fabry-Perot (F-P) cavity as a function of wavelength and SiO_2_ thickness. (**b**) Electric field intensity profiles of the designed blue (463 nm) and amber (575 nm) filters at the resonance peak wavelengths. (**c**) Comparison of the transmittance spectra of the filters with (w/) and without (w/o) SiO_2_ (10 nm) capping layer. (**d**) Measured (solid) and simulated (dash lines) transmittance spectra of the fabricated filters. Transmittance spectra (circles) of SU-8 interlayer is also shown.

The transmittance of the F-P cavity that has two interfaces is expressed by the following^39^:


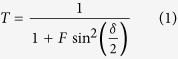


where 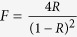, which is the coefficient of finesse and *R* is reflectance, and 
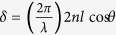
, which is the phase difference between successive transmitted light waves. *λ, n* and *l* are wavelength of incident light, refractive index and thickness of a medium, respectively. Multiple reflected lights between two reflecting surfaces allow constructive (destructive) interference to occur at a certain wavelength, which corresponds to a peak (dip) in the transmission, depending on the thickness and refractive index. To selectively transmit a desired wavelength range of visible light, we used a metal-insulator-metal (MIM) structure-based F-P cavity, which consisted of an optically transparent dielectric material (SiO_2_) separated by two semi-transparent thin silver (Ag) films (~29 nm) allowing light propagation for the design of transmission (color) filters. Figure 2a presents a transmission of the F-P cavity as a function of SiO_2_ thickness and wavelength at fixed Ag thickness of 29 nm calculated by transfer matrix method, showing that a transmission peak shifts from ~400 nm to ~800 nm covering the whole visible spectrum by increasing the thickness of SiO_2_ dielectric layer from ~78 nm to ~216 nm. We designed blue and amber color filters for our devices having their resonances (i.e., transmission peaks) at ~463 and ~575 nm, respectively, both of which corresponded to the SiO_2_ thickness of 100 nm and 140 nm, respectively. Figure 2b exhibits a distribution of the total electric field (E-field) intensities at those resonance wavelengths, presenting that the E-field is well confined in the dielectric layer between the two semitransparent Ag layers. As mentioned, a thin SiO_2_ capping layer (10 nm) was deposited on top of the F-P cavity to prevent the oxidation of the top Ag layer occurring during post-process, and we note that the effect of this protection layer on the optical properties of the F-P cavity is almost negligible as shown in Fig. 2c. The simulated transmission spectra of the proposed color filters on a glass substrate are provided in Fig. 2d, showing great agreement with measured profiles, both of which exhibit transmission efficiency higher than 65%; slight peak shifts to ~475 nm and ~582 nm were observed due to the process variation of e-beam evaporation. Transmittance of the SU-8 (~2 μm) layer, which was applied for an interlayer on top of the MoS_2_ phototransistor, over the measured spectrum range was about 90% as shown in Fig. 2d.

**Figure 3:**
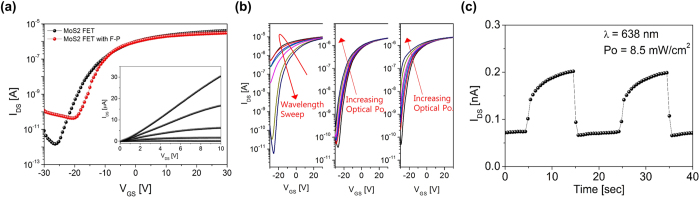
(**a**) Transfer characteristics of the as fabricated MoS_2_ field-effect transistor and after F-P cavity filter (amber) integration (Inset) Output characteristics for the filter integrated MoS_2_ phototransistor. (**b**) Transfer curves shifts for a wavelength sweep and optical power density sweep on the integrated MoS_2_ phototransistor: (left) wavelength sweep range of 430–790 nm with 60 nm steps, optical power density range of 0, 4, 8, 12, 16, 40, 64 mW/cm^2^ at (center) 638 nm and (right) 470 nm. (**c**) Photoswitching behavior of the integrated MoS_2_ phototransistor at V_DS_ = 1 V and V_GS_ = −25 V to light pulses with a period of 20 sec.

Figure 3a shows the electrical characteristics of a representative MoS_2_ phototransistor before and after F-P cavity integration. For the bare-MoS_2_ device, field-effect mobility (*μ*_*FE*_) of ~67.8 cm^2^/Vs in a linear operation region was extracted from


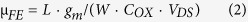


where *g*_*m*_ is the transconductance, *W*/*L* is the channel width/length, *C*_*OX*_ is PVP gate dielectric capacitance. The on/off current ratio of approximately 10^6^ was obtained. The *μ*_*FE*_ was maintained after the F-P integration (*μ*_*FE*_ ~64.4 cm^2^/Vs). However, the on/off current ratio was decreased by an order due to increased leakage current, which could be ascribed to additional charge generation and diffusion at the interface between the MoS_2_ and SU-8 interlayer. Due to thermal budget of the PET flexible substrate, high temperature annealing process was skipped after the SU-8 development, resulting in the SU-8 interlayer film not to be fully cross-linked^40^. As shown in the inset of Fig. 3a, output current did not saturate within the bias conditions, and exhibited good linearity at low *V*_*DS*_ bias indicating Ohmic-like contact behavior between MoS_2_ and Au electrode^41^,^42^.

The transfer-curve evolutions in Fig. 3b show the photoresponse of MoS_2_ phototransistor with the F-P cavity filter depending on (left) the swept wavelength range of 430–790 nm (60 nm step) and the incident optical power range of 0, 4, 8, 12, 16, 40, 64 mW/cm^2^ at both (center) 638 nm and (right) 470 nm. In the swept-wavelength measurement, a maximum transfer-curve modulation occurs around ~600 nm, which shift slightly compared to the resonance wavelength of integrated filter. While measuring the photoresponse under different illuminating wavelengths, a constant optical power density of 0.55 ± 0.15 mW/cm^2^ was maintained. Spectral responses will be discussed in the following. As for the power-dependent photoresponse, the transfer-curves shift gradually in response to the illuminating optical power densities. Those transfer-curve shifts (i.e. threshold voltage changes) indicate that the photoresponse is based on the photogating effect^43^. Figure 3c shows the photo-switching behavior of the MoS_2_ phototransistor integrated with the F-P cavity filter. We measured *I*_*DS*_ changes as the illuminating light (*λ*: 638 nm, Po: 8.5 mW/cm^2^) was turned on and off with a period of 20 sec. The observed identical response throughout the multiple cycles demonstrates stable and constant photoswitching performance of the integrated MoS_2_ phototransistor.

**Figure 4:**
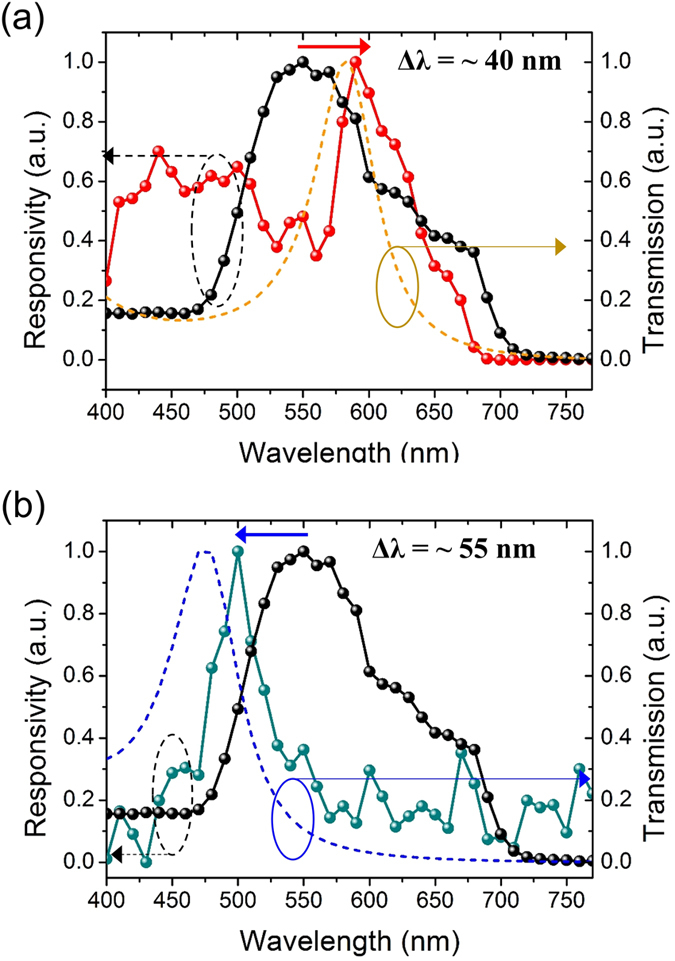
Spectral responsivities, which are normalized to the each measured maximum responsivity, are plotted for the flexible MoS_2_ phototransistors integrated with F-P cavity color filters for (**a**) amber (590 nm) and (**b**) blue (495 nm) wavelength ranges. The normalized spectral responsivity of the bare MoS_2_ phototransistor as well as normalized transmittance of the integrated F-P filters are also plotted alongside for comparison.

Spectral response characteristic of the bare MoS_2_ phototransistor (i.e. without a filter) was measured at *V*_*GS*_ = −28 V over the incident wavelength range of 400–780 nm using a tunable monochromatic light source, and its normalized responsivity to the maximum value is plotted in Fig. 4 (black lines). The spectrum reveals a sharp increase in response at *λ* ~690 nm, which is attributed to a direct band gap transition at the K-point of the Brillouin zone, and there was no observable responses via indirect band transitions at longer wavelengths. We also have observed two bumps at 610 and 660 nm on the right shoulder of the responsivity peak, corresponding to the exciton A and B absorption peaks^44^ (see Supplementary Fig. S1). Interestingly, the spectrum exhibits an unusual enhancement in response at *λ* ~550 nm, which can be ascribed to an optical resonance in the MoS_2_ layer. In the bare MoS_2_ phototransistor structure of Al/PVP/MoS_2_/air (50/400/108 nm), a weak light confinement in the MoS_2_ layer was confirmed based on the finite-difference time-domain (FDTD) simulation (see Supplementary Fig. S2), contributing the unexpected rise in the responsivity.

The spectral responsivity of the fabricated MoS_2_ phototransistors with the designed F-P cavity filters was measured in the same manner. The red line in Fig. 4a and the dark cyan line in Fig. 4b represent the normalized spectral responsivity for the devices with the amber and blue filters, respectively. The measured transmission characteristics of the designed filters were normalized to each peak transmission and plotted as dash lines in both figures. It is clearly observed that the responsivity spectra follow the transmission of the integrated filters, and therefore the detecting wavelengths can be tuned by integrating the F-P filter. The measured spectra have shifted peak responsivities at *λ* ~590 nm for the amber filter and *λ* ~495 nm for the blue filter compared to the bare MoS_2_ phototransistor. The discrepancies (amber: *Δλ* ~8 nm and blue: *Δλ* ~20 nm) between the resonant wavelengths of designed filter and the peak responsivity of the integrated phototransistor can be attributed to the thickness variation of the SiO_2_ layer; a small thickness variation of 3–4 nm corresponds to the shift of resonant wavelengths as depicted in Fig. 2a. It is to be noted that the spectral responses of the MoS_2_ phototransistor can be successfully controlled using the F-P cavity filter, indicating color-selectivity.

**Figure 5:**
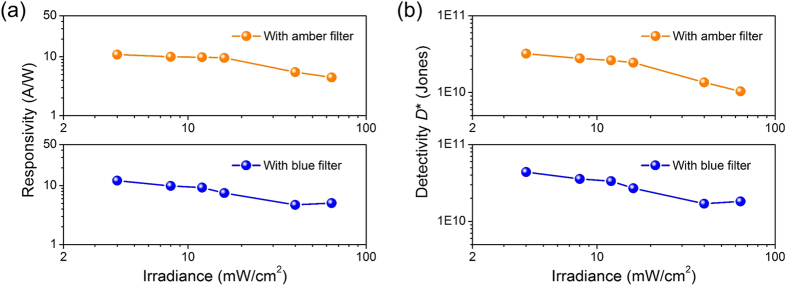
(**a**) Optical power-dependent responsivities of the flexible MoS_2_ phototransistors at a wavelength of 638 nm with optical power densities of 4, 8, 12, 16, 40, 64 mW/cm^2^ and applied V_DS_ = 1 V. (**b**) Specific detectivities (D^*^) as a function of the irradiance optical power densities were also plotted.

The power dependence of responsivity was measured and extracted at effective gate bias *V*_*GS*_ − *V*_*TH*_ = 5 V under illumination of 638 nm laser with a spot size of 300 μm. The upper and lower panel of Fig. 5a represent the power dependent responsivity of the MoS_2_ phototransistors with amber and blue filters, respectively. The responsivity remains constant at low incident power densities and then drops slightly as the incident power increases higher than ~10 mW/cm^2^. The photogating effect, as observed in the transfer-curves shifts, originates from charge trapping processes, and the overall responsivity will eventually decline as the number of photogenerated carriers at a high irradiance overwhelms the number of the induced carriers by a finite number of traps^27^ (see Supplementary Information Fig. S3). Given that the major contributor to the total current is the shot noise at dark current, specific detectivity (D*) is given by


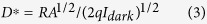


where *R* is the responsivity, *A* is the detecting area, *q* is the unit charge, and *I*_*dark*_ is the dark current. Figure 5b shows that the calculated detectivity exhibits a similar power-dependency in the range of 10^10^–10^11^ Jones. Still the detectivity can be improved by reducing the dark current after the SU-8 interlayer coating.

## Conclusions

In summary, we demonstrated flexible and color-selective MoS_2_ phototransistors with monolithically integrated transmission Fabry-Perot (F-P) cavity filters. The fabricated MoS_2_ transistors on the PAR substrate exhibit decent electrical characteristics (*μ*_*FE*_ > 64.4 cm^2^/Vs, on/off ratio > 10^6^) without much degradation after the SU-8 interlayer and F-P cavity integration. Our designed color filter were expected to cover whole visible range by varying the SiO_2_ thickness, and the integrated F-P filters successfully modulate the spectral response of MoS_2_ phototransistors from ~495 nm (blue) to ~590 nm (amber). The power dependence of both responsivity and specific detectivity shows similar trend with other reports, dominated by the photogating effect. By further optimizing the F-P cavity filter design and adopting mono- or few-layer MoS_2_ layer, we expect to further enhance the optical performance of the integrated MoS_2_ phototransistors. When combined with large-area MoS_2_ synthesis, our demonstration can be further developed into ultra-thin flexible photodetectors for wearables, conformable image sensor, and other optoelectronic applications.

## Methods

### Flexible MoS_2_ device fabrication

First, 200 μm thick polyarylate (PAR, A200HC) film was attached to a carrier glass substrate using an adhesive layer. Al (50 nm) gate-metal was deposited onto PAR by thermal evaporation, followed by PVP gate-dielectric formation; the PVP dielectric layer was formed by spin-coating at 3000 rpm for 60 sec and cross-linking process for 30 min at 150 °C. Following the dielectric formation, mechanically exfoliated MoS_2_ flakes from bulk MoS_2_ crystals (Graphene market, USA) by a conventional scotch-tape method were transferred onto the PVP layer. Then, Au (80 nm) as source/drain (S/D) electrodes was deposited by thermal evaporation and then patterned using conventional lift-off process. After integrating F-P cavity filters, the PAR film with the fabricated devices was released from the carrier glass. During the fabrication process, temperature was controlled below 150 °C to avoid thermal stress onto the PAR film and adhesive layer.

### Integration of Fabry–Perot (F-P) cavity filters

After MoS_2_ device fabrication, SU-8 (2000.5, MicroChem) was spin-coated over the devices twice for target thickness of ~2.5 um, and then S/D contact was opened by conventional exposure and develop process. Then the designed F-P cavity structure were deposited using e-beam evaporation as Cu (1 nm)/Ag (29 nm)/SiO_2_ (100 nm for blue; 140 nm for amber)/Ag (29 nm)/SiO_2_ (10 nm). Thin Cu layer was used to improve wettability of the thin Ag film on the SU-8 layer to minimize the light scattering loss induced from a rough surface. Resonance wavelengths of the F-P cavity are created by altering the thickness of the SiO_2_ layer at the fixed Ag thickness. Lastly, S/D contact areas were opened using wet chemical etching. The SiO_2_ capping layer was used to protect the top Ag layer from oxidation during S/D contact opening.

### Device characterization

3D laser scanning microscope (VK-X Series, Keyence) was used to obtain optical images and device dimensions (*W*/*L*) of the fabricated MoS_2_ phototransistors. Transmission spectra of the integrated color filter was measured by using a visible spectrophotometer (V-770 UV-Visible-Near Infrared Spectrophotometer, JASCO). Current-voltage (I-V) measurements were performed in an ambient condition using a semiconductor parameter analyzer (HP 4156 A). Power-dependent responsivity was measured under illumination with a spot size of 300 μm at a wavelength of 638 nm (Civillaser). Spectral response was measured by illuminating the device with fiber-coupled monochromatic light source consisting of 450 W Xe arc lamp and a monochromator.

## Additional Information

**How to cite this article:** Yoo, G. *et al*. Flexible and Wavelength-Selective MoS_2_ Phototransistors with Monolithically Integrated Transmission Color Filters. *Sci. Rep.* **7**, 40945; doi: 10.1038/srep40945 (2017).

**Publisher's note:** Springer Nature remains neutral with regard to jurisdictional claims in published maps and institutional affiliations.

## Supplementary Material

Supplementary Information
