# Supplementary material for: Flexible and Wavelength-Selective MoS2 Phototransistors with Monolithically Integrated Transmission Color Filters
Source: Sci Rep. 2017 Jan 18;7:40945. doi: 10.1038/srep40945 (PMC5241883; doi:10.1038/srep40945)
Supplement: Supplementary Information [file srep40945-s1.pdf]

Supplementary Information

Flexible and Wavelength-Selective MoS_2 Phototransistors with Monolithically Integrated Transmission Color Filters

Geonwook Yoo[1], Sol Lea Choi[2], Sang Jin Park[3], Kyu-Tae Lee[4], Sanghyun Lee[5], Min Suk Oh[2], Junseok Heo[5,*] and Hui Joon Park[3,5,*]

[1]School of Electronic Engineering,
Soongsil University, Seoul, 06938, South Korea

[2]Display Materials & Components Research Center,
Korea Electronics Technology Institute, Gyeonggi 13509, South Korea

[3]Department of Energy Systems Research,
Ajou University, Suwon 16499, South Korea

[4]Department of Materials Science and Engineering,
University of Illinois, Urbana-Champaign, Illinois 61801, USA

[5]Department of Electrical and Computer Engineering,
Ajou University, Suwon 16499, South Korea

1. Bandstructure of bulk MoS_2

The bare MoS_2 phototransistor in the manuscript has an approximately 108 nm thick MoS_2 layer which can be treated as a bulk. Figures S2 represents the bandstructure of bulk MoS_2 in which the direct bandgap transitions (A and B) at the K-point and indirect band transition (I) between the valence band maximum at Γ-point and the conduction band minimum are also indicated. Two excitonic responsivity peaks at 610 and 660 nm mentioned in the manuscript correspond to the exciton A and B transitions, respectively, as shown in the figure. This energy difference is due to the spin-orbit coupling of the valence band.

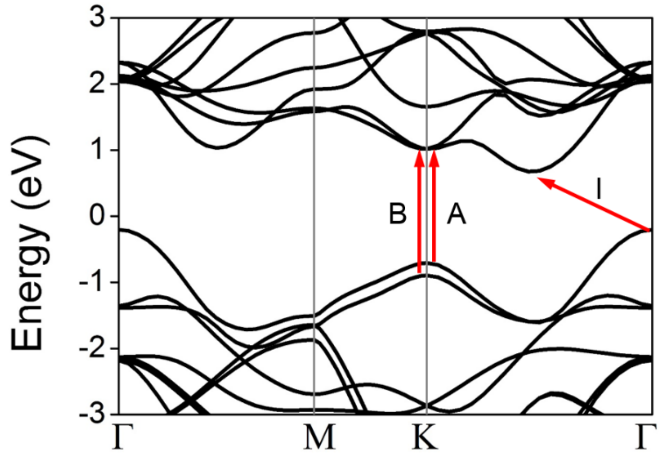

Figure S1. Bandstructure of bulk MoS_2.

2. Finite-difference time-domain (FDTD) simulation of the bare MoS_2 structure

We performed the finite difference time domain (FDTD) simulation to analyze the unusual enhancement of responsivity in the bare MoS_2 phototransistor. A stack of 50 nm-thick Al, 400 nm-thick PVP, and 108 nm-thick MoS_2 layers was considered for the simulation. The reflection spectrum was obtained by monitoring the reflected optical field at the MoS_2 surface when a plane wave was incident on the MoS_2 layer [Figure S1a]. The spectrum shows minimum reflectance at a wavelength of ~535 nm due to the resonance in the multilayered MoS_2 flake. On the other hand, we launched a plane wave source in the MoS_2 layer and monitored the field intensity inside as shown in Figure S1b. The large intensity of optical field at the identical wavelength (535 nm) was confirmed. Therefore, the multilayered MoS_2 is able to weakly confine incoming light at the resonant wavelength determined by the layer thickness. In result, this confinement modifies the responsivity spectrum of the bare MoS_2 phototransistor as mentioned in the paper.

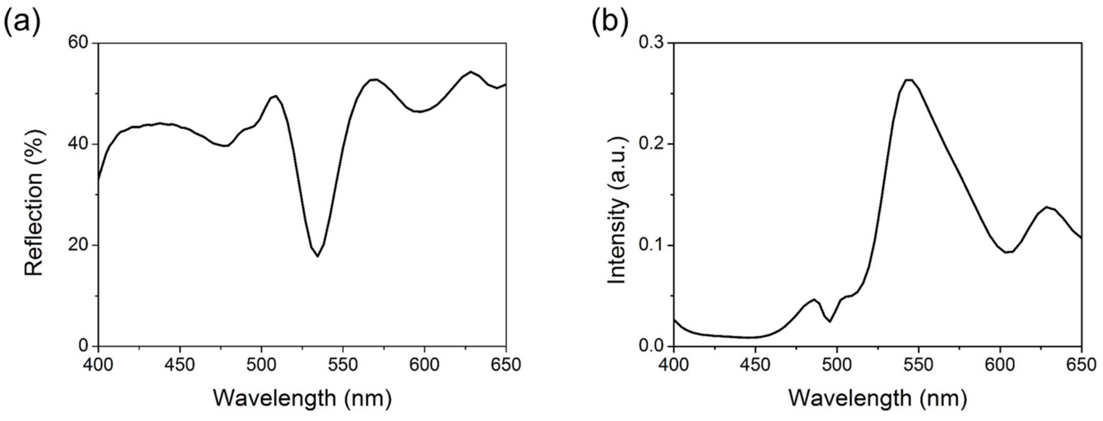

Figure S2. Finite-difference time-domain (FDTD) simulation of the bare MoS_2 structure (Al: 50nm/ PVP: 400 nm/ MoS_2: 108 nm) as fabricated in this work for (a) reflection spectra under normally incident light and (b) field intensity in the MoS_2 layer.

3. Carrier transport mechanism under different illumination

Under low illumination power densities, the responsivity remains constant and drops slightly as the incident power increases. The photoconductive gain is resulted from the carrier trapping and subsequent inducing of the opposite carriers to balance charge neutrality. Hence, when a weak light is illuminated, the long-lived deep hole traps are first filled and a long lifetime traps contribute a large photoconductive gain. On the other hand, the short-lived shallow traps are getting filled as the illumination intensity increases, and overall photoconductive gain is reduced. With further increase of the illumination intensity, the hole traps are eventually saturated and photoconductive gain is not expected anymore.

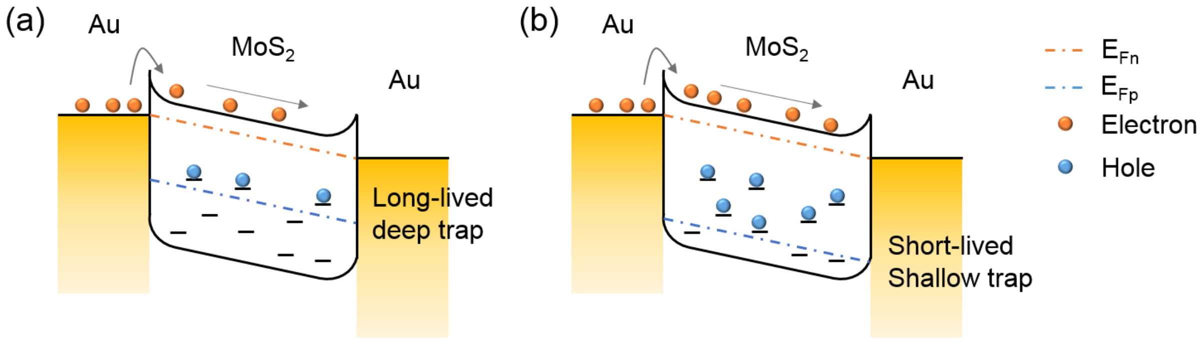

Figure S3. Carrier transport under weak illumination (a) and strong illumination (b). (a) Holes are trapped at long-lived deep hole traps. The long lifetime results in a large photoconductive gain. (b) Under strong illumination, the short-lived shallow traps are filled and overall photoconductive gain is reduced.
